# Assessing the Suitability of Next-Generation Viral Outgrowth Assays to Measure Human Immunodeficiency Virus 1 Latent Reservoir Size

**DOI:** 10.1093/infdis/jiaa089

**Published:** 2020-03-09

**Authors:** Mars Stone, Daniel I S Rosenbloom, Peter Bacchetti, Xutao Deng, Melanie Dimapasoc, Sheila Keating, Sonia Bakkour, Douglas D Richman, John W Mellors, Steven G Deeks, Jun Lai, Subul Beg, Janet D Siliciano, Amélie Pagliuzza, Nicolas Chomont, Carol Lackman-Smith, Roger G Ptak, Michael P Busch

**Affiliations:** 1 Vitalant Research Institute, San Francisco, California, USA; 2 Department of Laboratory Medicine, University of California, San Francisco, San Francisco, California, USA; 3 Department of Biomedical Informatics, Columbia University College of Physicians and Surgeons, New York, New York, USA; 4 Department of Epidemiology and Biostatistics, University of California, San Francisco, San Francisco, California, USA; 5 VA San Diego Healthcare System, San Diego, California, USA; 6 Center for AIDS Research, University of California, San Diego, La Jolla, California, USA; 7 Department of Medicine, University of Pittsburgh School of Medicine, Pittsburgh, Pennsylvania, USA; 8 Department of Medicine, University of California, San Francisco, School of Medicine, San Francisco, California, USA; 9 Department of Medicine, Johns Hopkins University School of Medicine, Baltimore, Maryland, USA; 10 Centre de Recherche du CHUM and Department of Microbiology, Infectiology and Immunology, Faculty of Medicine, Université de Montréal, Montreal, Quebec, Canada; 11 Southern Research, Frederick, Maryland, USA

**Keywords:** HIV reservoir, quantitative viral out growth assay (QVOA), assay comparison, IUPM, latency, leukapheresis, HIV cure, Inducible HIV RNA

## Abstract

**Background:**

Evaluations of human immunodeficiency virus (HIV) curative interventions require reliable and efficient quantification of replication-competent latent reservoirs. The “classic” quantitative viral outgrowth assay (QVOA) has been regarded as the reference standard, although prohibitively resource and labor intensive. We compared 6 “next-generation” viral outgrowth assays, using polymerase chain reaction or ultrasensitive p24 to assess their suitability as scalable proxies for QVOA.

**Methods:**

Next-generation QVOAs were compared with classic QVOA using single leukapheresis-derived samples from 5 antiretroviral therapy–suppressed HIV-infected participants and 1 HIV-uninfected control; each laboratory tested blinded batches of 3 frozen and 1 fresh sample. Markov chain Monte Carlo methods estimated extra-Poisson variation at aliquot, batch, and laboratory levels. Models also estimated the effect of testing frozen versus fresh samples.

**Results:**

Next-generation QVOAs had similar estimates of variation to QVOA. Assays with ultrasensitive readout reported higher infectious units per million values than classic QVOA. Within-batch testing had 2.5-fold extra-Poisson variation (95% credible interval [CI], 2.1–3.5-fold) for next-generation assays. Between-laboratory variation increased extra-Poisson variation to 3.4-fold (95% CI, 2.6–5.4-fold). Frozen storage did not substantially alter infectious units per million values (−18%; 95% CI, −52% to 39%).

**Conclusions:**

The data offer cautious support for use of next-generation QVOAs as proxies for more laborious QVOA, while providing greater sensitivities and dynamic ranges. Measurement of latent reservoirs in eradication strategies would benefit from high throughput and scalable assays.

## Teaser

We demonstrated similar estimates of variation for classic and next-generation quantitative viral outgrowth assays (QVOAs), with enhanced sensitivity supporting the use of next-generation QVOAs as a proxy for classic QVOAs in measuring the latent human immunodeficiency virus reservoir.

The human immunodeficiency virus (HIV) reservoir of latently infected cells is established early in infection and primarily exists in long-lived memory T cells, which have the capacity to be induced to produce new rounds of replication if suppressive anti-retroviral therapy (ART) is interrupted [1, 2]. This long-lived reservoir is a major barrier to cure. Accurate and scalable methods are needed to precisely measure the replication-competent reservoir, particularly to quantify changes in reservoir size to assess the efficacy of curative treatments [3–6].

To determine how successful an intervention has been to reduce the size of the latent reservoir, the quantitative viral outgrowth assay (QVOA) has been considered the reference standard [7]. However, there are limitations to our ability to accurately assess the latent reservoir using classic QVOA. The assay is resource and labor intensive, has significant cellular input requirements, requires potent stimulation of purified resting CD4^+^ cells in serial dilution coculture with repeated addition of target and feeder cells, and requires sensitive monitoring for the detection of HIV outgrowth. Furthermore, in vitro stimulation to reverse latency and induce outgrowth may be incomplete or insufficient and may not recapitulate the in vivo context of latency reversal. After 20 years of use, QVOA remains laborious, and only recently have there been rigorous assessments of its performance compared with other reservoir measurement strategies [8–10].

Next-generation HIV reservoir assays have been developed that are less labor intensive and costly and require lower input cell numbers, but this reduction in input cells and replicate well analyses could affect assay sensitivity and precision. In addition, increased sensitivity achieved by enhanced detection methods may not necessarily reflect intact and replication-competent virus [4, 11, 12].

In 2019, our group reported findings from a rigorous blinded panel study comparing results from 4 laboratories performing classic QVOAs [10]. In the current follow-up study, we sought to assess 3 things: the precision of next-generation QVOA under real experimental conditions, the effect of cryopreservation on infectious units per million (IUPM) values, and assess the suitability of next-generation assays as proxies for classic QVOA.

Even under perfect conditions, there is some inherent and unavoidable variability in measuring the HIV reservoir by IUPM outgrowth owing to Poisson sampling variation of a rare population of infected cells, resulting in relatively wide variation in target cells even between split samples from the same collection. To address sources of variation, the Reservoir Assay Validation and Evaluation Network (RAVEN) Study Group developed methods and models for statistical analysis of the accuracy and precision of QVOA in 75 split samples from 5 ART-suppressed participants using 4 classic QVOAs, and measured the impact of cryopreservation and laboratory-specific practices on assay results [10]. Variation at 3 levels was described: between split samples in the same testing batch, between batches tested with the same assay, and between laboratories performing different assays [10]. That study provided evidence for a lack of substantial systematic differences in IUPM measurement between fresh and frozen samples, supporting the use of frozen samples for batched analysis to monitor the impact of reservoir-reducing treatments before and after interventions and avoid the logistical difficulties in performing QVOA on fresh cells.

In the current investigation, we estimated the sources of variation that influence next-generation QVOA relative to classic QVOA results. We applied statistical methods to estimate the extra variation introduced by experimental conditions beyond that expected from Poisson variation, and to estimate the suitability of next-generation QVOA to serve as a proxy for classic QVOA.

## METHODS

### Study Design and Objectives

Assessing the suitability of next-generation assays as proxies for classic QVOAs requires considering that, within a large reservoir of cells containing HIV proviruses (HIV infected), a much smaller subset is measurable by QVOA [12–14]. Some fraction of this larger HIV-infected reservoir is clinically meaningful, but not the entire pool of HIV DNA-positive cells. Not all provirus-positive cells are replication competent; however, in vitro QVOAs may fail to measure all latently infected cells capable of expressing replication-competent HIV [12]. Next-generation assays arise from an attempt to develop more sensitive and less cumbersome methods to assess the clinically relevant and biologically meaningful pool of inducible infected cells. Using analytic methods developed in our assessment of classic QVOAs, we sought to understand how well several next-generation QVOAs correspond to classic QVOAs and to each other, and to assess the impact of freezing cells on next-generation assay performance.

### Experimental Design

Participants in the RAVEN project were enrolled and followed up as part of the University of California, San Francisco, OPTIONS and SCOPE studies, with specific consent for apheresis collections and testing for this study as approved by the University of California, San Francisco, Committee on Human Research (Institutional Review Board). Study design and participant characteristics (Supplementary Table 1) were described in detail elsewhere [10].

Five ART-suppressed HIV-1–infected participants based on time of initial ART after infection who have well-suppressed viral replication >3 years were included in the comparison; persons treated during acute infection (within 6 months) were excluded in order to have a reasonably established reservoir, increasing the potential for measurable and reproducible results. Specifically, participants were selected based on preexisting QVOA data to include participants with detectable and varying levels of previously characterized inducible virus, based on the Siliciano laboratory QVOA results, as reported by Eriksson et al [8] One HIV-uninfected control was included, and all 6 participants underwent leukapheresis collections of mononuclear cells. Isolated peripheral blood mononuclear cells from each collection were divided into identical replicate aliquots for testing in blinded fresh and frozen panels with multiple classic QVOA and next-generation assays. 

Five laboratories participated in the study and performed 9 HIV reservoir assays: University of Pittsburgh (QVOA M) [15], University of California, San Diego (QVOA RNA; inducible cell-associated RNA expression in dilution (iCARED) cell-free HIV RNA [cfRNA]/cell-associated HIV gag RNA [caRNA1]/cell-associated HIV tat-rev RNA [caRNA2]) [5, 9], Johns Hopkins University (QVOA S) [13, 16], Southern Research (QVOA SR and QVOA Simoa) [13, 16, 17], Centre de Recherche du CHUM (tat/rev-induced limiting dilution assay [TILDA]) [18]. All laboratories except Southern Research tested both fresh and frozen samples. Frozen panels of 18 uniquely blinded aliquots were batched to enable measurement of both within-batch and between-batch variation. Median estimate IUPM values with 97.5%, 95%, 90%, and 75% credible intervals (CIs) for each participant sample on each assay are summarized in Table 1.

**Table 1.** Median Estimate of Infectious Units per Million Values for Each Participant by Assay

Participant No. and Assay	IUPM Value								
	2.5% CI	5% CI	10% CI	25% CI	Median Estimate	75% CI	90% CI	95% CI	97.5% CI
Participant 1126									
QVOA M	0.62	0.69	0.81	1.07	1.44	1.91	2.56	3.03	3.44
QVOA RNA	4.63	5.47	6.26	8.19	10.98	15.35	20.06	22.93	26.61
QVOA S	0.42	0.49	0.58	0.80	1.18	1.63	2.19	2.63	3.10
QVOA SR	0.43	0.48	0.60	0.81	1.15	1.66	2.28	2.89	3.35
QVOA Simoa	2.44	2.86	3.30	4.51	6.43	9.27	13.07	15.76	18.69
TILDA	17.33	20.41	23.57	29.54	40.15	55.50	76.23	87.28	98.81
iCARED caRNA1	130.31	149.14	174.74	229.07	318.41	433.79	557.55	666.82	783.36
iCARED caRNA2	3.61	4.45	5.31	6.99	9.44	13.21	17.52	20.64	23.61
iCARED cfRNA	1.93	2.18	2.52	3.23	4.43	6.08	7.77	9.33	10.62
Participant 2026									
QVOA M	0.08	0.10	0.12	0.16	0.23	0.33	0.45	0.57	0.63
QVOA RNA	0.98	1.10	1.30	1.69	2.39	3.16	4.09	4.83	5.86
QVOA S	0.07	0.09	0.11	0.15	0.22	0.35	0.48	0.57	0.66
QVOA SR	0.02	0.03	0.04	0.05	0.09	0.14	0.20	0.25	0.30
QVOA Simoa	0.13	0.16	0.19	0.27	0.39	0.56	0.80	0.95	1.25
TILDA	2.94	3.30	3.93	5.14	6.93	9.63	12.47	15.13	16.72
iCARED caRNA1	9.83	11.02	12.99	17.04	23.61	31.87	43.31	48.64	57.18
iCARED caRNA2	1.07	1.17	1.38	1.82	2.48	3.37	4.58	5.20	6.05
iCARED cfRNA	0.54	0.60	0.74	1.01	1.40	1.93	2.55	3.03	3.43
Participant 2147									
QVOA M	0.18	0.21	0.26	0.35	0.47	0.64	0.87	1.00	1.15
QVOA RNA	1.97	2.22	2.66	3.47	4.69	6.47	8.59	10.55	12.15
QVOA S	0.31	0.36	0.43	0.57	0.82	1.12	1.51	1.81	2.18
QVOA SR	0.02	0.03	0.05	0.08	0.13	0.20	0.30	0.39	0.47
QVOA Simoa	0.16	0.19	0.22	0.33	0.52	0.78	1.17	1.44	1.66
TILDA	59.85	69.76	80.21	107.23	144.95	199.08	265.36	313.98	362.28
iCARED caRNA1	29.19	33.68	40.44	54.68	76.65	104.06	142.57	159.39	175.80
iCARED caRNA2	13.06	15.50	18.04	24.61	34.08	47.34	64.33	75.99	87.38
iCARED cfRNA	1.08	1.27	1.54	2.05	2.78	3.72	4.90	5.80	6.65
2208									
QVOA M	0.14	0.16	0.20	0.26	0.35	0.49	0.66	0.77	0.91
QVOA RNA	1.74	1.97	2.43	3.26	4.46	6.05	8.09	9.51	11.16
QVOA S	0.05	0.06	0.07	0.09	0.13	0.18	0.24	0.29	0.33
QVOA SR	0.02	0.03	0.04	0.06	0.09	0.13	0.18	0.23	0.30
QVOA Simoa	0.08	0.10	0.12	0.19	0.28	0.41	0.58	0.71	0.87
TILDA	9.34	11.33	13.65	19.33	27.65	40.05	56.07	69.75	83.06
iCARED caRNA1	25.20	29.07	34.70	45.98	60.65	82.80	106.38	126.91	148.59
iCARED caRNA2	4.45	5.41	6.30	8.00	10.96	15.15	19.20	22.55	25.34
iCARED cfRNA	0.33	0.38	0.45	0.59	0.80	1.12	1.48	1.76	1.92
Participant 3068									
QVOA M	0.16	0.19	0.24	0.32	0.46	0.66	0.90	1.07	1.27
QVOA RNA	1.43	1.59	1.93	2.53	3.56	4.81	6.39	7.78	9.22
QVOA S	0.09	0.10	0.12	0.17	0.26	0.38	0.55	0.66	0.76
QVOA SR	0.03	0.04	0.05	0.08	0.12	0.18	0.25	0.32	0.37
QVOA Simoa	0.21	0.26	0.30	0.40	0.59	0.87	1.20	1.50	1.73
TILDA	2.91	3.51	4.30	5.88	8.31	11.54	15.31	18.71	21.34
iCARED caRNA1	31.76	36.70	42.13	54.34	71.14	94.38	120.75	150.07	176.69
iCARED caRNA2	2.34	2.78	3.31	4.32	5.85	7.75	10.27	12.26	13.87
iCARED cfRNA	0.36	0.42	0.52	0.67	0.89	1.20	1.59	1.85	2.15

Abbreviations: caRNA1, cell-associated human immunodeficiency virus (HIV) gag RNA; caRNA2, cell-associated HIV tat-rev RNA; cfRNA, cell-free HIV RNA; CI, credible interval; iCARED, inducible cell-associated RNA expression in dilution; IUPM, infectious units per million; QVOA, quantitative viral outgrowth assay; QVOA M, QVOA by University of Pittsburgh; QVOA RNA, QVOA by University of California, San Diego, with HIV RNA readout; QVOA S, QVOA by Johns Hopkins University; QVOA Simoa, QVOA by Southern Research using Simoa readout; QVOA SR, QVOA by Southern Research; TILDA, tat/rev-induced limiting dilution assay.

### Analytical Methods

We used methods described elsewhere [10] to account for unavoidable Poisson variation and estimate additional sources of assay variation. The experimental design permitted us to identify 4 separate levels of random variation, which are as follows, from lowest to highest. First, additional variation may affect each aliquot of a split sample independently, even when the aliquots are measured by the same assay and in the same batch. Second, batch-to-batch variation may cause aliquots assayed in separate batches to tend to differ more than if they were assayed in the same batch. Third, a split sample with aliquots measured by 2 different assays may tend to differ more than if the aliquots were measured by the same assay. This extra variability may reflect differences in procedures (Table 2) that exist among assays of the same general type (ie, classic QVOA, enhanced sensitivity QVOA, or next-generation QVOA). We measured this variability after correcting for systemic scale difference between assays. Fourth, a split sample with aliquots measured by assays of different types may tend to differ more than if the aliquots were measured using 2 assays of the same type. This additional variability may reflect the fact that the different types of assays are targeting different entities (although the different entities may be substantially correlated). We used this primarily comparison to assess how much each assay tended to differ from the 3 classic QVOAs, beyond how much the 3 differ from one another.

**Table 2.** Characteristics of Participating Assays, Including Starting Material, Number of Replicates Assayed, Culture Conditions, and Culture Monitoring Readout

Assays by Assay Type	Starting Material	Dilution Replicates, No.	Dilutions and Cell Input at Each Dilution	Stimulation	Target Cells	ARV Culture Additions	Culture Duration	HIV Target to Monitor HIV-1 Outgrowth Culture Supernatant	Assay to Monitor HIV-1 Outgrowth Culture Supernatant	Measurement
Classic QVOA										
QVOA M [15]	120 mL of WB; 300 × 10^6^ PBMCs	6	3-Fold 1 × 10^6^–1 × 10^2^ rCD4^+^	PHA plus γ-irradiated PBMCs	CD8-depleted PBMCs	None	2 wk	p24	PerkinElmer HIV-1 p24 ELISA	IUPM
QVOA S [7, 13]	≥100mL of WB; 400 × 10^6^ PBMCs	5–22	5-Fold 1 × 10^6^–0 rCD4^+^	PHA plus γ-irradiated PBMCs	MOLT-4/ CCR5	None	3 wk	p24	PerkinElmer HIV-1 p24 ELISA	IUPM
QVOA SR [7, 13]	≥100 mL of WB; 400 × 10^6^ PBMCs	5–22	5-Fold 1 × 10^6^–0 rCD4^+^	PHA plus γ-irradiated PBMCs	CD8-depleted PBMCs	None	20 d	p24	PerkinElmer HIV-1 p24 ELISA	IUPM
usQVOA										
QVOA Simoa [7, 13, 16]	≥100 mL of WB; 400 × 10^6^ PBMCs	5–22	5-Fold 1 × 10^6^–0 rCD4^+^	PHA plus γ-irradiated PBMCs	CD8-depleted PBMCs	None	20 d	p24	Quanterix p24 Simoa	IUPM
QVOA RNA [5, 9]	100 mL of WB; 7–20 × 10^6^ rCD4^+^	6	3-Fold 1 × 10^6^–1372 rCD4^+^	Plate-bound αCD3/ αCD28	MOLT-4/ CCR5	None	9–16 d	*gag*	RT-PCR	IUPM
Next-generation QVOA										
TILDA [17]	10–20 mL of WB; 10 × 10^6^ PBMCs	≤24	4-Fold 18 000 –1000 CD4^+^	PMA/ ionomycin	None	Zidovudine, efavirenz, raltegravir	18 h	Tat/rev msHIV caRNA	Nested RT-PCR	Inducible msHIV RNA
iCARED [5, 9] cfRNA, caRNA1, caRNA2	100 mL of WB; 7–20 × 10^6^ rCD4^+^	6–12	3-Fold 5 × 10^5^–229 rCD4^+^	Plate-bound αCD3/ αCD28	None	Raltegravir	3 d	Cell-associated and cell-free *gag*, tat-rev RNA	dd-PCR	Transcription- competent virus, single round infection

Abbreviations: ARV, antiretroviral therapy; caRNA, cell-associated RNA; caRNA1, cell-associated human immunodeficiency virus (HIV) gag RNA; caRNA2, cell-associated HIV tat-rev RNA; cfRNA, cell-free HIV RNA; dd-PCR, digital droplet polymerase chain reaction; ELISA, enzyme-linked immunosorbent assay; iCARED, inducible cell-associated RNA expression in dilution; IUPM, infectious units per million; MOLT-4, T-lymphoblastic cell line; msHIV, multiply spliced HIV; PBMCs, peripheral blood mononuclear cells; PHA, phytohemagglutinin; PMA, Phorbol 12-myristate 13-acetate; QVOA, quantitative viral outgrowth assay; QVOA M, QVOA by University of Pittsburgh; QVOA RNA, QVOA by University of California, San Diego, with HIV RNA readout; QVOA S, QVOA by Johns Hopkins University; QVOA Simoa, QVOA by Southern Research using Simoa readout; QVOA SR, QVOA by Southern Research; rCD4+, resting CD4+; RT-PCR, reverse-transcription polymerase chain reaction: TILDA, tat/rev-induced limiting dilution assay; usQVOA, QVOA with ultrasensitive detection HIV-1 outgrowth in culture supernatant; WB, whole blood.

These 4 levels of variation are modeled as random effects (normally distributed on natural log scale, centered at 0), whereas systemic assay scale differences and frozen storage were both modeled as fixed effects. We used Markov chain Monte Carlo methods to obtain posterior medians and 95% CIs for all effects. In addition to modeling all 9 assays together, we compared estimates obtained by modeling subsets separately: the 3 classic QVOAs, the 2 enhanced -sensitivity QVOAs, the 4 next-generation QVOAs, and the 3 classic QVOAs plus each of the other 5 assays (sets of 4 assays modeled together). Finally, we modeled every possible pair of different assays (36 different sets of 2) to obtain the most direct estimates of variation between pairs of assays. Each model assumed that the aliquot and batch sources of variability, along with the effect of frozen storage, were the same for all assays in the model. For reporting, random effect sizes (standard deviations) were exponentiated to obtain fold increases in variation.

We used the above estimates to project a “bottom line” expected error in each next-generation or enhanced sensitivity assay, relative to the classic QVOAs. Specifically, median absolute error (in log_10_ terms) was computed, as in reference 9, from each model that included the 3 classic QVOAs plus 1 other assay. A range of IUPM values on the QVOA M scale was assumed, and the error estimates were adjusted for the fixed scale differences, so those differences did not count as error for any assay. We excluded batch-to-batch variation in these calculations, on the assumption that it could be avoided in practice by selecting samples to be run in the same batch. Variation due to assay type was assigned entirely to the alternative assay, reflecting an assumption that the classic QVOAs measure the most relevant biological entities.

## RESULTS

### IUPM Values for RNA and Digital p24-Based Versus p24 Enzyme-Linked Immunosorbent Assays 

**Figure 1.**
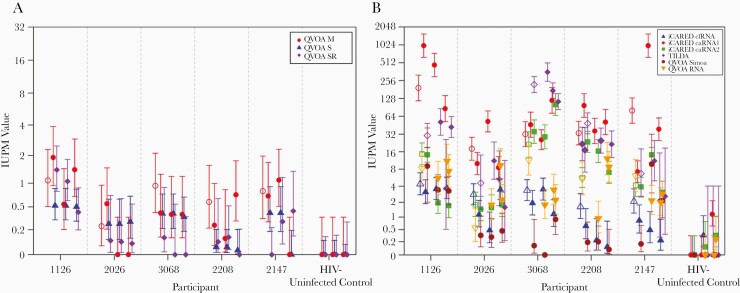
Maximum likelihood estimates and 95% credible intervals of infection frequency for each aliquot for classic (*A*) and next-generation and ultrasensitive-readout (*B*) quantitative viral outgrowth assay (QVOA). Solid symbols indicate cryopreserved aliquots; open symbols, fresh aliquots. Participants include 5 with human immunodeficiency virus (HIV) infection and 1 uninfected control. Abbreviations: caRNA1, cell-associated HIV gag RNA; caRNA2, cell-associated HIV tat-rev RNA; cfRNA, cell-free HIV RNA; iCARED, inducible cell-associated RNA expression in dilution; IUPM, infectious units per million; QVOA M, QVOA by University of Pittsburgh; QVOA RNA, QVOA by University of California, San Diego, with HIV RNA readout; QVOA S, QVOA by Johns Hopkins University; QVOA Simoa, QVOA by Southern Research using Simoa readout; QVOA SR, QVOA by Southern Research; TILDA, tat/rev-induced limiting dilution assay.

**Figure 2.**
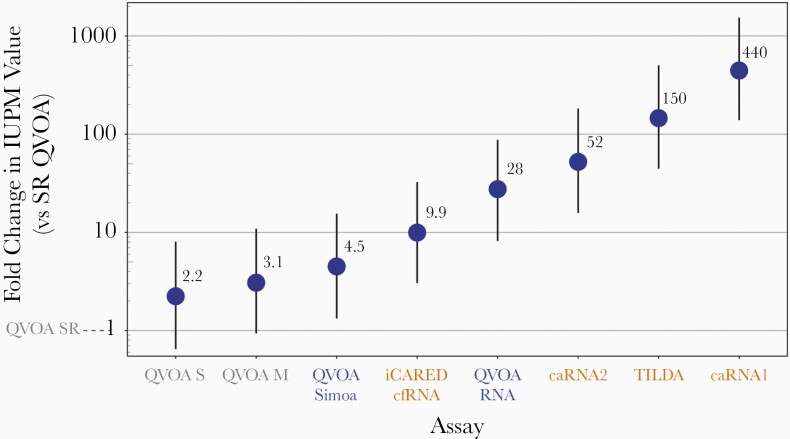
Median differences with 95% credible intervals between assays presented as fold differences in scale of typical output, in infectious units per million (IUPM), for 2 classic and 6 next-generation quantitative viral outgrowth assays (QVOAs; including QVOA with ultrasensitive readout), normalized to QVOA by Southern Research (QVOA SR). The estimated fold change used the model of all 9 assays together. Classic QVOAs are shown in gray, enhanced sensitivity assays in blue, and next-generation QVOAs in orange. Abbreviations: caRNA1, cell-associated human immunodeficiency virus (HIV) gag RNA; caRNA2, cell-associated HIV tat-rev RNA; cfRNA, cell-free HIV RNA; iCARED, inducible cell-associated RNA expression in dilution; QVOA M, QVOA by University of Pittsburgh; QVOA RNA, QVOA by University of California, San Diego, with HIV RNA readout; QVOA S, QVOA by Johns Hopkins University; QVOA Simoa, QVOA by Southern Research using Simoa readout; TILDA, tat/rev-induced limiting dilution assay.

The IUPM maximum likelihood estimates and 95% CIs of infection frequency for each aliquot are presented in Figure 1. Assays using polymerase chain reaction or digital p24 antigen (Ag) (Simoa) assays to detect ex vivo–induced virus demonstrated consistently higher IUPM values than classic QVOA, specifically, standard-sensitivity p24 Ag enzyme-linked immunosorbent assay (ELISA) (Figure 2). QVOA SR was chosen as the reference because it is a classic QVOA with p24 Ag ELISA readout and also tended to have the lowest values. Relative to typical QVOA SR, the 2 other p24 Ag ELISA-based assays (QVOA S and QVOA M) average 2.2- and 3.1-fold higher values, respectively. The 2 QVOAs using ultrasensitive detection (usQVOA) methods (QVOA Simoa and QVOA RNA) average 4.5- and 28-fold higher IUPM values. Next-generation QVOAs reported 4.5–444-fold higher IUPM values. At these higher levels of detection, it is important to note the possibility of detecting p24 Ag or RNA produced by replication-defective virus [19], although approximately half of such cells producing RNA are replication competent [20].

### Random Variation Between QVOA Types

Random variation was observed at the aliquot, batch, and assay levels, even after correction for systematic effects between assays and unavoidable Poisson error (Tables 3 and 4). When all 9 assays were considered together, aliquots tested in different batches using the same assay had 2.3-fold excess variation (95% CI, 2.0–2.7-fold). Split aliquots tested using different assays varied 3.1-fold (2.6–3.9-fold) beyond Poisson variation and systematic assay differences. Combined aliquot plus batch variation was estimated to be lower for the 3 classic QVOAs than for the other 2 types of assay, but CIs did overlap (1.9-fold variation for classic QVOA ultra sensitive vs 2.7-fold and 2.6-fold variation, respectively, for QVOA and next-generation QVOA).

**Table 3.** Estimated Extra-Poisson Variation and Effect of Cryopreservation After Adjustment for Systematic Assay Scale Differences Between 3 Assay Groups, at the Aliquot, Batch, and Assay Levels

Level of Analysis	Estimated Extra-Poisson Variation, Posterior Median (95% CI), Fold Change			
	3 Assay Groups (n = 9 Assays)	Classic QVOAs (n = 3)	Classic QVOAs (RNA/ Digital p24) (n = 2)	Next-Generation QVOAs (n = 4)
Aliquot level	2.2 (1.9–2.6)	1.6 (1.0–2.4)	1.0 (1.0–1.8)	2.5 (2.1–3.5)
Batch level (alone)	1.2 (1.0–1.8)	1.4 (1.0–2.5)	2.7 (1.8–4.9)	1.0 (1.0–1.9)
Aliquot + batch	2.3 (2.0–2.7)	1.9 (1.4–3.0)	2.7 (1.9–5.0)	2.6 (2.1–3.6)
Assay level (alone)	2.1 (1.6–2.9)	1.5 (1.0–2.8)	1.7 (1.0–9.8)	2.1 (1.0–4.0)
Aliquot + batch + assay	3.1 (2.6–3.9)	2.2 (1.5–3.9)	3.4 (2.1–12.9)	3.4 (2.6–5.4)
Assay type	1.1 (1.0–2.2)	NA	NA	NA
Aliquot + batch + assay + assay type	3.2 (2.6–4.3)	NA	NA	NA
Frozen effect, %	−22 (−47 to 13)	−37 (−77 to 51)	−22 (−77 to 175)	−18 (−52 to 39)

Abbreviations: CI, credible interval; NA, not applicable; QVOAs, quantitative viral outgrowth assays. Classic QVOA: QVOA employing standard sensitivity p24 Ag ELISA; Classic QVOAs (RNA/Digital p24): QVOA employing ultrasensitive RNA or digital readout; Next-Generation QVOA: inducible viral outgrowth assay.

**Table 4.** Estimated Extra-Poisson Variation for Effect of Cryopreservation for Classic Quantitative Viral Outgrowth Assay Versus Next-Generation Assays, After Adjustment for Fixed Scale Differences Between Assays at the Aliquot, Batch, and Assay Levels

Level of Analysis	Estimated Extra-Poisson Variation, Posterior Median Fold Change (95% CI), for Classic QVOA Versus Next-Generation Assays					
	QVOA RNA	QVOA Simoa	TILDA	caRNA1	caRNA2	cfRNA
Aliquot level	1.5 (1.1–2.2)	1.4 (1.0–2.0)	1.6 (1.3–2.2)	2.6 (2.1–3.4)	1.9 (1.6–2.5)	1.7 (1.4–2.1)
Batch level (alone)	1.7 (1.0–2.4)	2.1 (1.3–3.4)	1.2 (1.0–2.0)	1.0 (1.0–1.8)	1.2 (1.0–2.1)	1.2 (1.0–1.9)
Aliqot + batch	2.0 (1.6–2.7)	2.3 (1.6–3.6)	1.7 (1.4–2.5)	2.6 (2.2–3.5)	2.1 (1.7–2.8)	1.8 (1.5–2.3)
Assay level (alone)	1.5 (1.0–2.5)	1.4 (1.0–2.8)	2.0 (1.0–4.0)	1.5 (1.0–2.7)	2.0 (1.0–3.9)	1.4 (1.0–2.3)
Aliquot + batch + assay	2.3 (1.8–3.4)	2.5 (1.8–4.3)	2.5 (1.7–4.6)	2.9 (2.3–4.4)	2.8 (1.9–4.9)	2.0 (1.6–2.9)
Assay type	1.0 (1.0–2.4)	1.0 (1.0–2.9)	1.0 (1.0–7.1)	1.0 (1.0–3.0)	1.0 (1.0–5.8)	1.0 (1.0–2.3)
Aliquot + batch + assay + assay type	2.4 (1.8–4.0)	2.7 (1.8–5.0)	3.1 (2.1–8.0)	3.0 (2.3–5.5)	3.2 (2.3–7.6)	2.0 (1.6–3.5)
Frozen effect, %	−31 (−62 to 30)	−41 (−80 to 71)	−27 (−60 to 26)	−8 (−58 to 96)	−12 (−52 to 63)	−52 (−72 to −22)

Abbreviations: caRNA1, cell-associated human immunodeficiency virus (HIV) gag RNA; caRNA2, cell-associated HIV tat-rev RNA; cfRNA, cell-free RNA; CI, credible interval; NA, not applicable; QVOA, quantitative viral outgrowth assay; QVOA RNA, QVOA by University of California, San Diego, with HIV RNA readout; QVOA Simoa, QVOA by Southern Research using Simoa readout; TILDA, tat/rev-induced limiting dilution assay.

### Effect of Cryopreservation on IUPM Values

Overall, cryopreservation caused small reductions in IUPM values, but increases or an absence of an effect could not be ruled out. In the primary models tested (Tables 3 and 4), the estimated systematic fixed effect of cryopreservation on IUPM measurements involved reductions of 18%–37%, and all 95% CIs spanned the absence of an effect.

### Assay Log_10_ Error Comparisons

At low IUPM values, next-generation assays with higher readout scales tended to have smaller typical errors (median absolute log_10_ errors) than QVOA M and QVOA SR, owing to detection of more abundant targets (0.1 IUPM) (Supplementary Table 2). They did not similarly outperform QVOA S on this metric because of the high cell input, which is due to the high number of replicates performed in this QVOA. Differences in error diminished with increasing IUPM values, with the exception of iCARED caRNA1, which lost accuracy relative to other assays at high IUPM values; this is potentially attributable to a higher likelihood of all positive replicate wells at many dilutions and consequently a higher scale factor (typical IUPM output, 444-fold above QVOA SR) (Figure 2).

### Pairwise Comparison of Variation Between Assays

**Figure 3.**
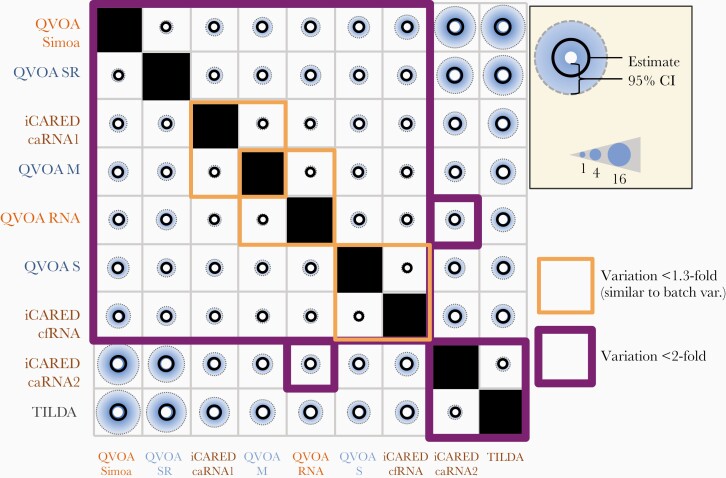
Between-assay random effect (fold variation in infectious units per million [IUPM] values). Comparison of extra-Poisson variation in split samples tested by different assays. Purple boxes indicate pairs of assays with <2-fold excess random variation; orange boxes, pairs with <1.3-fold excess variation; black circles, median estimates; blue shaded areas, upper limit of credible interval; and size of white center, lower limit of credible interval. One-fold variation is the minimum possible, corresponding to no excess variation after correction for any systematic scale effects (Figure 2). The fold variation in IUPM values is the result of exponentiating the standard deviation of the random effect modeled on the natural log scale. Abbreviations: caRNA1, cell-associated human immunodeficiency virus (HIV) gag RNA; caRNA2, cell-associated HIV tat-rev RNA; cfRNA, cell-free HIV RNA; iCARED, inducible cell-associated RNA expression in dilution; QVOA, quantitative viral outgrowth assay; QVOA M, QVOA by University of Pittsburgh; QVOA RNA, QVOA by University of California, San Diego, with HIV RNA readout; QVOA S, QVOA by Johns Hopkins University; QVOA Simoa, QVOA by Southern Research using Simoa readout; QVOA SR, QVOA by Southern Research; TILDA, tat/rev-induced limiting dilution assay.

Seven of the 9 assays studied had correlated readouts (random variation between all pairs, <2-fold) (Figure 3). These assays are all 3 classic QVOAs, both alternate-readout QVOAs, and both next-generation inducible QVOAs using gag templates (iCARED caRNA1 and cfRNA). Within this group, 3 pairs had particularly good agreement with each other (magnitude of between-assay variation not exceeding that of batch variation): iCARED caRNA1 and QVOA M, QVOA RNA and QVOA M, and iCARED cfRNA and QVOA S. The 2 other assays, TILDA and iCARED caRNA2, which both detect multiply spliced tat/rev transcripts, clustered together (random variation, <2-fold).

## Discussion

Intervention strategies designed to reduce or eliminate the latent reservoir require accurate, reproducible, and scalable assays to detect changes in reservoir size and estimate the efficacy of eradication efforts. Understanding the precision of assays in the context of latency-reducing interventions is critical both to assessing how well changes in latent reservoirs can be measured and how to design clinical study protocols to estimate reduction in reservoir size with desired precision. The classic QVOA, though historically the reference standard, is not scalable for routine use in clinical studies of latent reservoir–reducing interventions. In addition, the low readout scale of classic QVOA limits its sensitivity and dynamic range, and it is not routinely practical to obtain the larger peripheral blood mononuclear cell inputs needed to improve these assay characteristics. The greater sensitivity of next-generation assays reduces cell input requirements and increases the available dynamic range for measuring reductions in the size of the latent reservoir.

Although these assays measure related targets—CD4^+^ cells harboring inducible provirus-derived p24 Ag or RNA—there is variation among experimental approaches, even within assay categories [21, 22]. Even among classic QVOA procedures, there are differences in cell stimulation methods, feeder cell type, and even isolation of CD4^+^ cells (thus determining input cell numbers) [5, 6, 9, 23]. Because of these differences, each assay measures a slightly different aspect of latency, reflected in both the systematic and the random variation observed between assays [4, 14].

A single, complete measurement of the clinically relevant latent reservoir remains elusive [24]. Although the classic QVOA provides an underestimate, it is considered the best approximation, pending further progress. If systematic differences in IUPM measurements are quantified between types of assays (ie, replication-competent virus that is detectable as exponential progressive increases in supernatant p24 Ag detected by ELISA in classic QVOA vs induced virus supernatant or cell-associated HIV Ag or RNA detected by next-generation assays), then the more scalable next-generation assays could be used as proxies for classic QVOA, capitalizing on their enhanced sensitivity, relative precision, and dynamic range.

Once systematic differences in assay scale were accounted for, we found that next-generation assay readout both correlated with classic QVOA and exhibited similar levels of random variation. In some cases, the excess variation associated with using a next-generation assay as proxy for classic QVOA was found to be similar to that of batch-to-batch variation (Figure 3). This finding provides initial evidence that some next-generation assays may in fact be suitable proxies. It remains to be seen, however, whether responses to latency-reducing agents or other therapeutic interventions are similar across assays.

In experimental practice, combined aliquot and batch variation may be more relevant than variation at a single level, because aliquot- and batch-level variation would be combined when samples are tested in different batches. In theory, if a research study could batch samples from a participant, then only aliquot variation (the first source of excess variation described in Methods) would contribute to extra variability above Poisson variability. Batch size may be limiting because most laboratories cannot set up large batches, this might not be a factor if one needs to assay only 2–3 longitudinal samples from a participant to measure the efficacy of an intervention.

Assays using ultrasensitive means of monitoring QVOA culture supernatants tend to report approximately 4.5–28-fold higher IUPM values than classic QVOA when normalized to QVOA SR (Figure 2). The increased sensitivity of monitoring outgrowth by RNA or digital p24 Ag assays may come at the cost of the inability to distinguish clinically relevant virus that is capable of robust replication from defective or ineffective virus or nonpackaged viral RNA [4]. However, it has been recently shown that approximately half of the cell-free virions measured in the more sensitive QVOA RNA assay are replication competent [20], suggesting that the efficient propagation of these virions in culture may be a limiting step contributing to underestimation of the size of the reservoir given by cell culture–based assays. 

Interestingly, newer modifications of the QVOA can increase the sensitivity by as much as 20-fold [25]. In addition, cells that produce HIV Ag, but not replication-competent virus, may merit clinical attention as contributors to immune activation and pathogenesis [26]. Cells carrying defective proviruses can produce viral RNA; therefore, higher IUPM values with RNA-based assays may reflect cells with defective proviruses or suboptimal efficiency of QVOAs. Only by demonstrating exponential increases in viral RNA over time can these assays demonstrate replication-competent virus.

In the current study, we found that cryopreservation had a <2-fold effect on IUPM estimates. The cryopreservation process, however, is complex and quality of the procedure can vary dramatically in different study sites. It is important to note that experienced researchers carried out all laboratory procedures, and thus our results likely represent a best-case scenario. Our analysis also assumes that freezing causes a fixed fold change in all samples. If cryopreservation had opposite effects on different subsets of the reservoir, it could decrease assay reliability in a manner not captured by our study.

Outgrowth assays using culture-based methods tend to underestimate the true size of the latent reservoir, and measurement of outgrowth depends on the capacity of infected cells to produce infectious virus on stimulation [5, 22]. Further work is needed to clarify the genetic nature of HIV provirus and induced virions and thus the replication capacity of infected cells producing cell-associated and/or cell-free RNA. This should include genetic characterization of low-level virus observed in cultures lacking the robust kinetics required for detection with classic QVOA monitored by p24 ELISA. 

Specifically, it will be important to determine whether induced viral RNA or virus with low-level growth kinetics at concentrations not detectable by ELISA are replication competent, and thus relevant to the reservoir that would rebound when treatment is interrupted. This would be informed by genetic characterization of the HIV transcripts detected and virus present at low levels in culture wells, and by assessment of the intactness of provirus producing such transcripts and virions. Preliminary studies have shown that examination of longitudinal outgrowth kinetics and single-genome sequencing analyses verified replication competence of reactivated virus in some cases [25]. However, what is critically needed for the field is to identify, evaluate, and validate methods that could accurately predict the time to rebound after treatment interruption. Although beyond the scope of this comparison study, development of a consortium similar to the RAVEN program to collaborate with therapeutic trials involving treatment interruption is needed to inform future eradication strategies.

Overall, our results offer cautious support for applying next-generation assays with systematically higher readouts as proxies for the more laborious and less sensitive classic QVOA. Analytical tools introduced by Rosenbloom et al [10]. allow rigorous comparison of outgrowth-based dilution coculture and next-generation polymerase chain reaction assays that are designed to specifically quantify intact proviruses or transcripts, pointing the way toward precise, efficient assessments of HIV cure strategies [26–28]. The RAVEN program is now executing larger-scale studies evaluating many of these assays.

## Supplementary Data

Supplementary materials are available at *The Journal of Infectious Diseases* online. Consisting of data provided by the authors to benefit the reader, the posted materials are not copyedited and are the sole responsibility of the authors, so questions or comments should be addressed to the corresponding author.

jiaa089_suppl_Supplementary-Table-1

jiaa089_suppl_Supplementary-Table-2

## Notes

***Acknowledgments.*** The Reservoir Assay Validation and Evaluation Network (RAVEN) Study Group Steering Committee includes the following members: M. P. B., Silvija Staprans, J. M., D. D. R., S. D., P. B., Joe Fitzgibbon, and R. G. P; the Central Laboratory staff include M. S., S. K., S. B., and X. D; and the key collaborators were Ron Bosch, Rebecca Ho, Robert Siliciano, J. S., N. C., D. I. S. R., and Sulggi Lee.

***Financial support.*** This work was supported by the Bill & Melinda Gates Foundation (Collaboration for AIDS Vaccine Discovery grant INV-008500 to the RAVEN Study Group); amfAR, The Foundation for AIDS Research (Mathilde Krim fellowship 109511-61-RKRL to D. I. S. R.); the Division of AIDS, National Institute of Allergy and Infectious Disease, National Institutes of Health (contract HHSN272201500017C, supporting work at Southern Research); the National Institutes of Health (grants 1UM1AI126619, through the Delaney Collaboratories for AIDS Research on Eradication, and 1UM1Al126620, through DEAT-HIV, both to D. R.); the San Diego Center for AIDS Research (grant AI306214 to D. R.); the Department of Veterans Affairs (D. R.); the James B. Pendleton Charitable Trust (D. R.); the Delaney AIDS Research Enterprise to Find a Cure (grant UM1AI126611 to N. C.); and the Quebec Health Research Fund (research scholar career award grant 253292 to N. C.).

***Potential conflicts of interest.*** All authors: No reported conflicts. All authors have submitted the ICMJE Form for Disclosure of Potential Conflicts of Interest. Conflicts that the editors consider relevant to the content of the manuscript have been disclosed.

**Presented in part:** DAIDS/NIAID Strategies for HIV Cure Meeting, Bethesda, MD, 14–16 November 2016; and Conference for Retroviruses and Opportunistic Infections, Boston, MA, 4–7 March 2018.
